# miR-491-5p, mediated by Foxi1, functions as a tumor suppressor by targeting Wnt3a/*β*-catenin signaling in the development of gastric cancer

**DOI:** 10.1038/cddis.2017.134

**Published:** 2017-03-30

**Authors:** Ruifang Sun, Zhigang Liu, Dongdong Tong, Yang Yang, Bo Guo, Xiaofei Wang, Lingyu Zhao, Chen Huang

**Affiliations:** 1 Department of Pathology, School of Basic Medical Sciences, Xi'an Jiaotong University Health Science Center, 76 Yanta West Road, Xi'an, Shaanxi 710061, P.R. China; 2 Key Laboratory of Environment and Genes Related to Diseases, Xi'an Jiaotong University, Ministry of Education of China, 76 Yanta West Road, Xi'an, Shaanxi 710061, P.R. China; 3 Department of Surgical Oncology, The First Affiliated Hospital of Xi'an Jiaotong University, 277 Yanta West Road, Xi'an, Shaanxi 710061, P.R. China; 4 Department of Thoracic Surgery, Shaanxi Provincial Tumor Hospital, Xi'an Jiaotong University, 309 Yanta West Road, Xi'an, Shaanxi 710061, P.R. China; 5 Department of Cell Biology and Genetics, School of Basic Medical Sciences, Xi'an Jiaotong University Health Science Center, 76 Yanta West Road, Xi'an, Shaanxi 710061, P.R. China; 6 School of Public Health, Xi'an Jiaotong University Health Science Center, 76 Yanta West Road, Xi'an, Shaanxi 710061, P.R. China; 7 Key Laboratory of Shaanxi Province for Craniofacial Precision Medicine Research, College of Stomatology, Xi'an Jiaotong University, 76 Yanta West Road, Xi'an, Shaanxi 710061, P.R. China

## Abstract

Accumulated evidence has suggested that microRNAs (miRNAs) have an important role in tumor development and progression by regulating diverse signaling pathways. However, the precise role of miRNAs in gastric cancer (GC) has not been elucidated. In this study, we describe the function and regulation network of miR-491-5p in GC. miR-491-5p is frequently downregulated in GC tissues compared with adjacent non-cancerous tissues. Forced expression of miR-491-5p significantly inhibits proliferation and colony formation, and promotes apoptosis in GC cells. Through bioinformatic analysis and luciferase assays, we confirm that miR-491-5p targets Wnt3a. Silencing Wnt3a inhibits cell proliferation and induces apoptosis. Similarly, restoration of Wnt3a counteracts the effects of miR-491-5p expression. Moreover, bioinformatic and luciferase assays indicate that the expression of miR-491-5p is regulated by Foxi1, which binds to its promoter and activates miR-491-5p expression. In conclusion, to the best of our knowledge, our findings are the first to demonstrate that Foxi1 is a key player in the transcriptional control of miR-491-5p and that miR-491-5p acts as an anti-oncogene by targeting Wnt3a/*β*-catenin signaling in GC. Our study reveals that Foxi1/miR-491-5p/Wnt3a/*β*-catenin signaling is critical in the progression of GC. Targeting the pathway described in this study may open up new prospects to restrict the progression of GC.

Gastric cancer (GC) is the fourth most frequent malignant disease and the second most common cause of cancer-related deaths worldwide.^1^ GC is an aggressive tumor that is generally diagnosed at an advanced stage. Although much progress has been made in treatment, the combination of therapy strategies has not contributed to a significant impact on the clinical outcome of patients with advanced GC.^2^ An increasing number of studies have focused on the exploration of molecular mechanisms underlying the development of GC, which may provide a foundation for novel therapeutic targets.^3^

MicroRNAs (miRNAs) are a class of small non-coding nucleotides (18–25 nucleotides in length) that can negatively regulate target gene expression, and they function as tumor promoters or suppressors. These small molecules often bind to 3′-untranslated regions (3′-UTR) of messenger RNA (mRNA) by suppressing translation, or by degrading mRNA according to complete or incomplete complementarity.^4^ Among these miRNAs, miR-491-5p, located in the fourth intron of focadhesin (FOCAD) has been reported to be deregulated in several human cancers.^5, 6, 7, 8, 9^ miR-491-5p acts as a tumor suppressor by targeting JMJD2B in ERa-positive breast cancer.^10^ miR-491-5p and its targeted gene GIT1 are considered biomarkers for the prognosis of oral squamous cell carcinoma.^11^ miR-491-5p has been shown to inhibit metastasis, and epithelial to mesenchymal transition in hepatocellular carcinoma and esophageal cancer by suppressing matrix metalloproteinase (MMP) or targeting protein for xenopus kinesin-like protein 2 (TPX2), respectively.^12, 13^ Besides, upregulation of miR-491-5p suppressed proliferation and induced apoptosis of the human osteosarcoma cells.^14^ Our present study shows that miR-491-5p is markedly decreased in GC tissues compared to non-cancerous adjacent tissues, suggesting that miR-491-5p may act as a tumor suppressor in GC.

miRNAs function as tumor suppressors or oncogenes by directly targeting specific genes.^15^ On the basis of the literature reviews and gene target prediction databases, including TargetScan (http://www.targetscan.org/), miRanda (http://www.microrna.org/), and miRBASE (http://www.mirbase.org/), we hypothesized that Wnt3a may be a potential target of miR-491-5p. The *Wnt3a* gene is an important member of the Wnt ligand family, which exerts its function by activating the canonical Wnt signaling pathway.^16^ When Wnt signaling is activated, Wnt ligand binds to its receptor frizzled (Fz) and co-receptor lipoprotein receptor-related protein (LRP5/6). This binding boosts the stabilization of *β*-catenin in the cytoplasm, inducing it to translocate into the nucleus where it forms a ternary complex with T-cell-specific transcription factor (TCF)/lymphoid enhancer-binding factor (LEF) followed by transcriptional activation of target genes, such as cyclin D1 and c-myc.^17^ Wnt3a is overexpressed in advanced prostate cancer cells, which activates transcription factor (ATF3)-induced tumors.^18^ Wnt3a is highly expressed in most breast cancer cell lines^19^ and oral squamous cell carcinoma.^20^ However, in mouse or human melanoma cell lines, Wnt3a overexpression prohibits proliferation *in vitro* and tumor growth *in vivo*.^21^ Moreover, Wnt3a inhibits the growth and proliferation of the lung and lacrimal glands,^22^ induces cell death of B-cell precursor acute lymphoblastic leukemia (B-ALL), and inhibits the proliferation of several B-ALL cell lines,^23^ These findings suggests that Wnt3a may have varied effects on different cancer types. A previous study has shown that Wnt3a is highly expressed in human scirrhous gastric carcinoma 44As3 cells with highly metastatic derivatives.^24^ The expression pattern of Wnt3a in GC tissue and the underlying molecular mechanisms remain to be further studied.

To the best of our knowledge, the upstream regulator of miR-491 remains to be clarified. It has been recognized that transcription factors, epigenetic modification, and the processing of pri-miR to mature miRNAs could play important roles in miRNA expression regulation.^25^ Thus, the potential regulation of miR-491-5p by transcription factors needs to be investigated. Bioinformatic databases, including UCSC genome browser tool, VISTA (http://genome.lbl.gov/cgi-bin/WGRVistaInput5.pl?cfg_dir=gp_r4099_169), and JASPAR database (http://jaspar.genereg.net/) was applied and indicated the presence of a Foxi1-binding region in the promoter of the *miR-491* gene. Foxi1, also known as HFH3, belongs to the forkhead family, and the specific function of this gene has not yet been determined. However, it is possible that Foxi1 plays an important role in the development of the cochlea and vestibular duct as well as embryogenesis.^26, 27, 28^ Thus, it needs to be clarified if Foxi1 mediates miR-491-5p expression and plays a role in the development of GC.

The aim of the present study was to explore the function and underlying mechanism, including the upstream transcription factor and downstream target gene of miR-491-5p, in GC carcinogenesis. We provide evidence that Foxi1 mediates miR-491-5p and plays a crucial role in the regulation of proliferation and apoptosis of GC cells via Wnt3a/*β*-catenin signaling pathways. Our data were obtained based on gain- and loss-of-function studies of Foxi1, miR-491-5p, and Wnt3a *in vivo* and *in vitro*. To our knowledge, this is the first study that directly illustrates a Foxi1/miR-491-5p/Wnt3a axis in GC.

## Results

### miR-491-5p inhibits cell proliferation of GC *in vitro*

**Figure 1:**
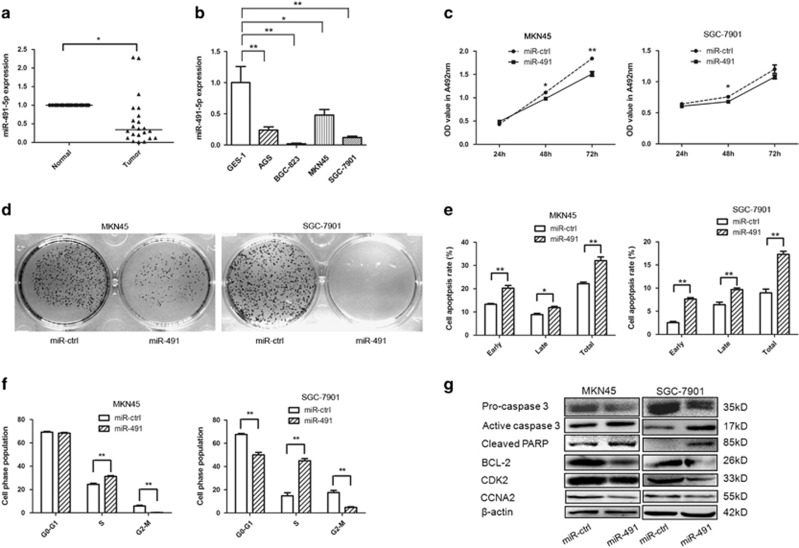
miR-491-5p inhibits cell proliferation and promotes apoptosis in gastric cancer cells. (**a**) qRT-PCR was performed to examine miR-491-5p expression in 22 paired human gastric cancer and adjacent normal tissues. The expression of miR-491-5p was normalized to U6. (**b**) qRT-PCR analysis of miR-491-5p expression in normal gastric mucosal and gastric cancer cells and normalized against U6 RNA. (**c**) The effects miR-491-5p on gastric cancer cell proliferation were determined by MTT assay after transfection of miR-491 or control vector at 24, 48, and 72 h. (**d**) Representative results of a colony formation assay for MKN45/SGC-7901 cells after miR-491 transfection. (**e**) Apoptosis was detected by annexin-V/propidium iodide combined labeling flow cytometry in MKN45/SGC-7901 cells 48 h after transfection with miR-491 or control vector. Apoptotic evaluation was carried out by calculating the percentage of apoptotic cells. (**f**) MKN45/SGC-7901 cells were transfected with miR-491 vector and control vector. After 48 h, cell cycle distribution was analyzed by flow cytometry. A histogram indicates the percentage of cells in G0/G1, S, and G2/M cell cycle phases. (**g**) The expression of pro-caspase 3, active caspase 3, cleaved PARP, BCL-2, CDK2, CCNA2, and *β*-catenin was analyzed by western blot (*P<0.05, **P<0.01)

We analyzed the expression pattern of miR-491-5p in 22 pairs of GC tissue and matched adjacent non-cancerous tissue samples using qRT-PCR. Compared to normal tissues, miR-491-5p was significantly downregulated in GC samples (Figure 1a). This result was also validated in four GC cell lines. miR-491-5p expression was decreased in SGC-7901, MKN45, BGC823, and AGS cell lines compared to the GES-1 cell line (Figure 1b). To clarify the function of miR-491-5p in GC, MKN45 and SGC-7901 cells were selected for further analyses.

**Figure 2:**
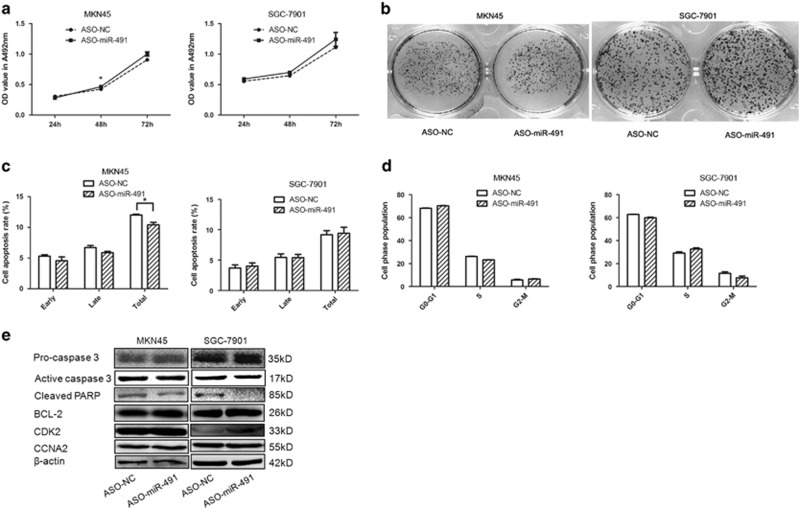
Inhibiting miR-491-5p contributes to gastric cell growth and apoptosis. (**a**) MTT assay of MKN45/SGC-7901 cells after transfection with ASO-miR-491 or a negative control. (**b**) The growth of MKN45/SGC-7901 cells was detected by colony formation after transfection with ASO-miR-491 or a negative control. (**c**) Apoptosis was determined in MKN45/SGC-7901 cells transfected with ASO-miR-491 or a negative control. (**d**) Cell cycle was determined in MKN45/SGC-7901 cells transfected with ASO-miR-491 or a negative control. (**e**) The expression of pro-caspase 3, active caspase 3, cleaved PARP, BCL-2, CDK2, CCNA2, and *β*-catenin was analyzed by western blot (*P<0.05, **P<0.01)

To explore the function and role of miR-491-5p, gain- and loss-of-function analyses were conducted. The expression level of miR-491-5p was markedly increased in the pre-miR-491-transfected cells compared to the control vector-transfected cells (Supplementary Figure S1A); the inhibitory effect was moderate due to the low expression of endogenous miR-491-5p in MKN45/SGC-7901 cells (data not shown). MTT and colony formation assays were applied, and the results showed that overexpression of miR-491-5p in MKN45 and SGC-7901 cells exerted an inhibitory effect on cell growth and colony formation after transfection (Figures 1c and d), while the miR-491-5p inhibitor exerted moderate adverse effects on GC cells that may be caused by the low expression level of miR-491-5p in MKN45/SGC-7901 cells (Figures 2a and b). The cell growth inhibitory effect of miR-491-5p was also performed by MTT assay in GES-1 cells (Supplementary Figure S1B). These data indicated that miR-491-5p may act as a tumor suppressor in GC.

### miR-491-5p induces apoptosis and cell cycle arrest in GC cells

To elucidate the mechanism of miR-491-5p modulation of GC cell growth, we tested if miR-491-5p affects apoptosis or cell cycle. Overexpression of miR-491-5p induced cell apoptosis in MKN45/SGC-7901 cells (Figure 1e). Moreover, miR-491-5p induced S/M phase arrest, upregulation of miR-491-5p led to an accumulation of the S-phase population with a reduction of the M phase population in MKN45/SGC-7901 cell lines compared to the negative control (Figure 1f). However, the miR-491-5p inhibitor exhibited a slight difference in cell apoptosis and no significant difference compared to cells transfected with the negative control, which may be due to the low expression level and low inhibition efficiency of miR-491-5p in MKN45/SGC-7901 cells (Figures 2c and d). We also evaluated the related inhibitory effect of miR-491-5p on apoptosis and cell cycle in GES-1 cells (Supplementary Figures S1C and D).

To investigate the underlying mechanisms of miR-491-5p in apoptosis and cell cycle regulation, we measured the expression levels of apoptosis- and cell cycle-related proteins in GC cells. miR-491 transfection in MKN45/SGC-7901 cells downregulated pro-caspase 3, BCL-2, CDK2, and CCNA2, but upregulated active caspase 3 and cleaved PARP (Figure 1g). In contrast, these protein expression levels exhibited opposite trends after miR-491-5p inhibitor transfection (Figure 2e and Supplementary Figure S1E). These findings demonstrate that miR-491-5p is overexpressed in GC cells and inhibits cell growth by leading to cell cycle arrest and inducing apoptosis in GC cells.

### Wnt3a is a direct functional target of miR-491-5p in GC

We searched for potential miR-491-5p target genes using computer-aided miRNA target prediction programs. Wnt3a was selected as a candidate because it has a potential miR-491-5p-binding site in its 3′-UTR. Furthermore, Wnt3a has been reported to be upregulated in human cancers, and involved in tumor growth and metastasis.^23, 29^

**Figure 3:**
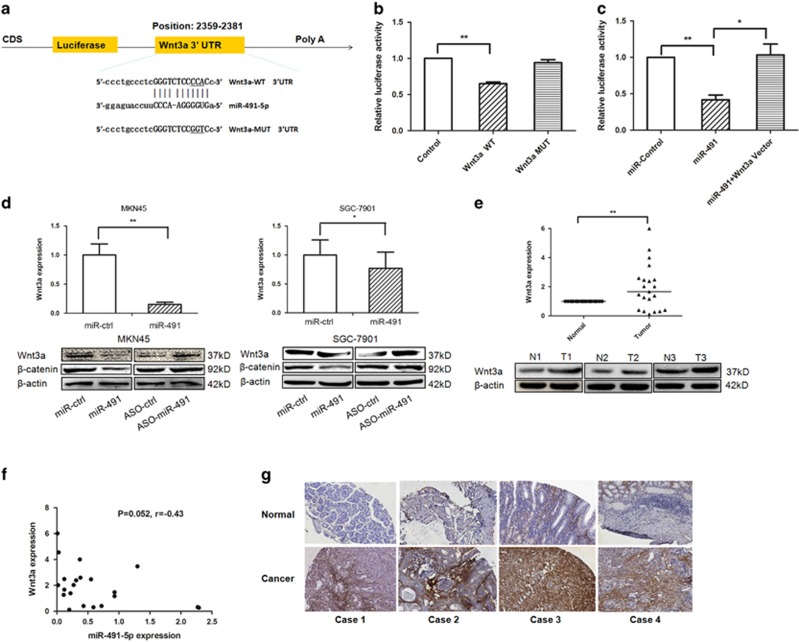
Wnt3a is a direct target of miR-491-5p in gastric cancer cell lines. (**a**) miR-491-5p is highly conserved across species and it has binding sites within the 3′-UTR of human Wnt3a. (**b**) The luciferase assay was performed in HEK293 cells in which miR-491 was co-transfected with pGLO-Wnt3a wild-type or pGLO-Wnt3a mutant vector. (**c**) The TCF/LEF reporter luciferase assay was performed in HEK293 cells in which pGL4.49 vector containing a TCF–LEF response element was co-transfected with miR-control, miR-491, and miR-491+Wnt3a vectors. (**d**) mRNA and protein expression levels of Wnt3a were measured by qRT-PCR (upper panel) and western blot (lower panel) after transfection with miR-491, ASO-miR-491, or a negative control in MKN45/SGC-7901 cells. (**e**) The expression levels of Wnt3a were evaluated by qRT-PCR and western blot. (**f**) The correlation between miR-491-5p and Wnt3a was analyzed. (**g**) Wnt3a protein expression levels were measured by IHC in gastric cancer tissue microarray (*P<0.05, **P<0.01)

To determine if miR-491-5p directly targets Wnt3a, we subcloned 3′-UTR Wnt3a fragments including wild-type (Wnt3a-WT) and mutant (Wnt3a-MUT) miR-491-5p-binding sites into the pmiRGLO dual-luciferase reporter vector (Figure 3a). pre-miR-491 and Wnt3a-WT- or MUT-3′-UTR vectors were co-transfected into HEK293 cells. The relative luciferase activity of the Wnt3a-WT pmirGLO-3′-UTR vector was significantly reduced in miR-491-overexpressing HEK293 cells. As expected, miR-491-5p failed to inhibit the luciferase activity of Wnt3a-MUT pmirGLO-3′-UTR vector, indicating that miR-491-5p binds directly to the 3′-UTR of Wnt3a (Figure 3b). In accordance with this result, the luciferase activity of TCF/LEF luciferase reporter was significantly decreased in miR-491 transfection group compared with miR-negative control transfection group, but not in miR-491+Wnt3a vector co-transfection group (Figure 3c). The same luciferase assays were also conducted in MKN45/SGC-7901 cells (Supplementary Figures S2A and B). This result further confirmed that miR-491 regulates Wnt3a/*β*-catenin signaling pathway by targeting the 3′-UTR of Wnt3a in the development of GC. In addition, when pre-miR-491, the miR-491-5p inhibitor and related controls were transfected into MKN45 and SGC-7901 cells, miR-491-5p decreased the expression of Wnt3a and its downstream target – *β*-catenin, and upregulated Wnt3a and *β*-catenin protein levels were observed when miR-491-5p was knocked down (Figure 3d). The expression levels of Wnt3a were significantly increased in gastric tissues compared to matched adjacent non-cancerous tissue in 22 pairs of tissues. Western blot data also confirmed the increased expression of Wnt3a in GC tissues (Figure 3e). Meanwhile, miR-491-5p levels were inversely correlated with Wnt3a expression by qRT-PCR assay (Figure 3f). Taken together, our data demonstrated that Wnt3a is a direct target of miR-491-5p.

We also examined Wnt3a expression levels by immunohistochemistry (IHC) using a tissue microarray of 92 pairs of GC and matched normal tissues. The clinicopathological features of the patients are summarized in Supplementary Table S1. Wnt3a was upregulated in 64.8% cancer tissues compared to normal tissues (Figure 3g). Overexpression of Wnt3a was significantly associated with a higher rate of perineural invasion in GC patients (*P*=0.022, Table 1).

### Knockdown of Wnt3a reduces the progression of GC cells and overexpression of Wnt3a eliminates the effects of miR-491-5p

**Figure 4:**
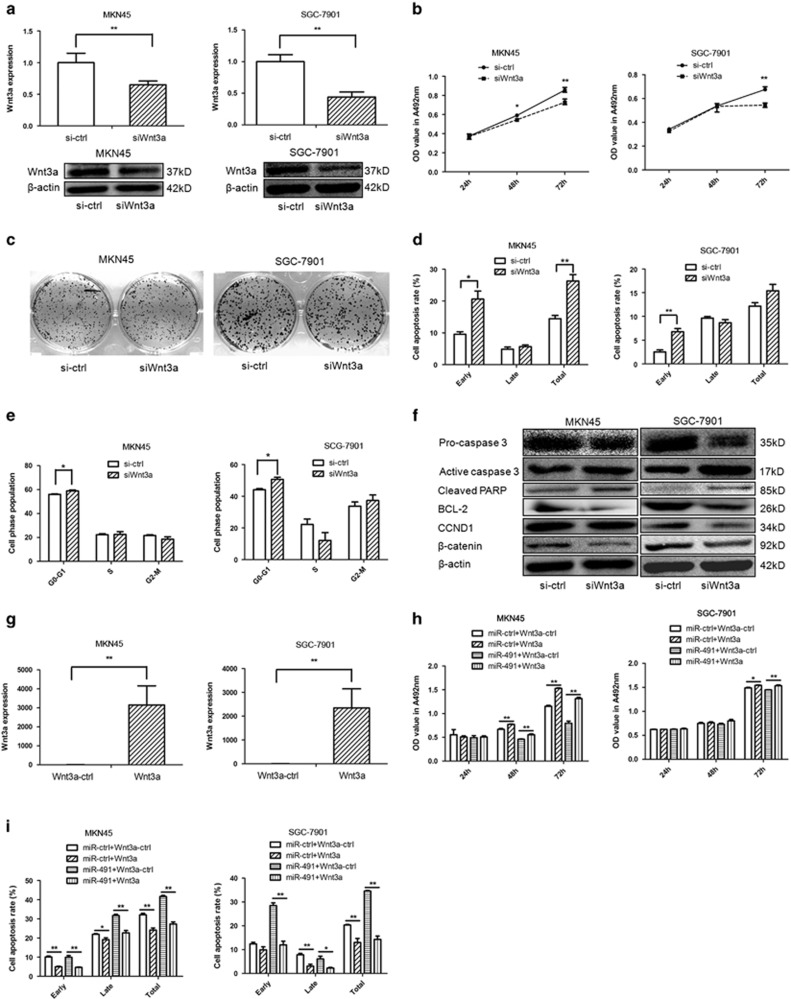
Expression levels of Wnt3a affect gastric cancer cell progression. (**a**) The expression levels of Wnt3a were measured by qRT-PCR (upper panel) and western blot (lower panel) in MKN45/SGC-7901 cells transfected with siWnt3a. (**b**) MTT assay was performed to determine the growth of gastric cancer cells treated with siWnt3a or a negative control (si−ctrl). (**c**) The colony formation assay was performed several days after transfection of gastric cancer cells with siWnt3a or a negative control (si−ctrl). (**d**) Apoptosis was determined in gastric cancer cells at 48 h after transfection with siWnt3a. (**e**) Cell cycle distribution was determined in gastric cancer cells 48 h after transfection with siWnt3a by propidium iodide staining and flow cytometry. The histogram indicates the percentage of cells in G0/G1, S, and G2/M cell cycle phases. (**f**) Protein expression of pro-caspase 3, active caspase 3, cleaved PARP, BCL-2, CCND1, and *β*-catenin in gastric cancer cells transfected with siWnt3a or si−ctrl was analyzed by western blot. (**g**) The expression levels of Wnt3a were determined by qRT-PCR in MKN45/SGC-7901 cells transfected with Wnt3a expression vector. (**h**) MTT assay was performed to measure the impact of gastric cancer cells treated with miR-491 plus Wnt3a expression vectors or related negative controls. (**i**) Cell apoptosis were performed to determine the effect of gastric cancer cells treated with miR-491 plus Wnt3a expression vectors or related negative controls (*P<0.05, **P<0.01)

We knocked down Wnt3a expression by RNA interference (small interfering RNA (siRNA)) to confirm that Wnt3a is implicated in the antitumor effects of miR-491-5p. Our results showed that Wnt3a was knocked down by siRNA both at the mRNA and protein levels (Figure 4a). Similar to miR-491-5p-overexpressing cells, downregulation of Wnt3a significantly inhibited proliferation and slightly inhibited colony formation in MKN45/SGC-7901 cells (Figures 4b and c). Moreover, knockdown of Wnt3a induced apoptosis in MKN45/SGC-7901 cells similar to the effect of miR-491-5p overexpression (Figure 4d). However, the influence of Wnt3a siRNA on cell cycle was not similar to miR-491-5p upregulation (Figure 4e). According to western blot analysis, Wnt3a siRNA decreased the expression of *β*-catenin, pro-caspase 3, BCL-2, and CCND1, and it increased the expression of active caspase 3 and cleaved PARP (Figure 4f). These findings indicated that miR-491-5p can regulate GC progression by directly targeting Wnt3a.

To further demonstrate that miR-491-5p inhibits tumor progression through targeting Wnt3a, we constructed rescue experiments. The expression levels of Wnt3a were markedly higher after transfection of the Wnt3a expression vector into MKN45/SGC-7901 cells (Figure 4g). Co-overexpression of Wnt3a and miR-ctrl or miR-491 in MKN45/SGC-7901 cells showed that Wnt3a overexpression reduced the tumor-suppressing effect of miR-491-5p on GC cell proliferation (Figure 4h). Moreover, Wnt3a decreased the cellular apoptotic effect of miR-491-5p in MKN45/SGC-7901 cells (Figure 4i). Our results showed that the effect of Wnt3a upregulation was eliminated by adding miR-491-5p to MKN45/SGC-7901 cells. These results provided additional evidence that miR-491-5p exhibits tumor suppressor role by directly targeting Wnt3a.

### MiR-491-5p induces growth inhibition of SGC-7901 cells *in vivo*

**Figure 5:**
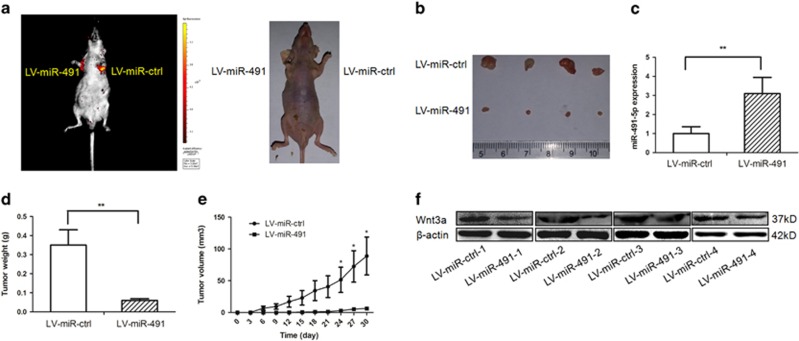
miR-491-5p inhibits gastric cancer progression *in vivo*. (**a**) At day 30, tumor growth was measured by *in vivo* bioluminescence imaging. The armpits were injected with SGC-7901 cells infected with LV-miR-ctrl (left armpit) and SGC-7901 cells infected with LV-miR-491 (right armpit) in four nude mice, respectively. (**b**) The gross morphology of tumors. (**c**) The expression levels of miR-491-5p were analyzed by qRT-PCR analysis in the tumor tissues from the animals. (**d**) Tumor weight was measured. (**e**) Tumor growth curves of tumor volume were formed every 3 days for 30 days (*n*=4). (**f**) The expression levels of Wnt3a were analyzed by western blot in tissues from the animals (*P<0.05, **P<0.01)

To further validate the tumor growth suppression role of miR-491-5p, we performed xenograft assays. miR-491-expressing SGC-7901 cells and negative control cells were injected subcutaneously into lateral armpits of mice. As expected, miR-491-5p played an important role in inhibiting tumor growth *in vivo*. Tumor nodules derived from miR-491-5p-overexpressing cells grew substantially less than those of the control group (Figures 5a and b). qRT-PCR confirmed significantly increased levels of miR-491-5p in xenograft tumors overexpressing miR-491 (Figure 5c). The tumor nodule weight was significantly less in miR-491-treated tumors than that of negative control-treated tumors (Figure 5d). On day 30, the average volume of miR-491-treated tumors was much less than that of control tumors (Figure 5e). As expected, *in vivo* data showed that the expression of Wnt3a in tumor tissues was reduced in miR-491-treated tumors by western blot (Figure 5f). These findings were consistent with the *in vitro* results and indicated that miR-491-5p has an anti-growth ability in GC by targeting Wnt3a *in vivo*.

### Foxi1 activates miR-491-5p expression

**Figure 6:**
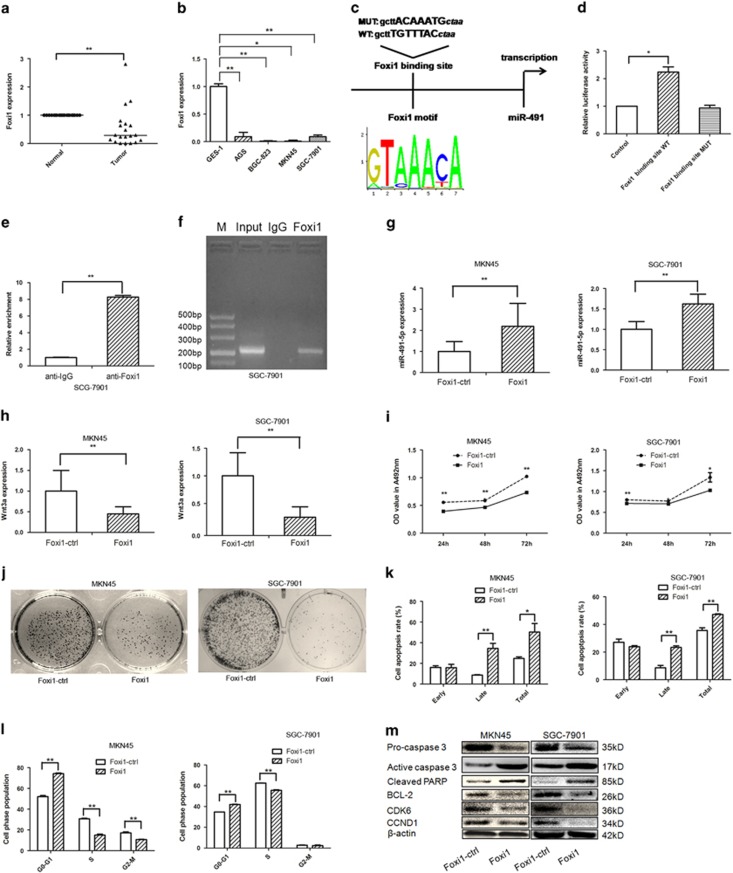
Foxi1 induces miR-491-5p promoter activity in gastric cancer cells. (**a**) The expression levels of Foxi1 mRNA in gastric cancer tissues were analyzed by qRT-PCR. (**b**) qRT-PCR analysis of Foxi1 expression in normal gastric mucosal and gastric cancer cells and normalized against U6 RNA. (**c**) Schematic diagram of the putative miR-491 promoter with one potential Foxi1 response element. (**d**) Luciferase activity of reporter constructs spanning the putative Foxi1-binding site or a negative control sequence. (**e**) ChIP assays were performed with control (rat IgG), anti-Foxi1 antibody to determine Foxi1 occupancy of miR-491 promoter. (**f**) qRT-PCR analysis was performed with primers spanning predicted Foxi1-binding site of miR-491 promoter. (**g**) The expression of miR-491-5p was assayed by qRT-PCR after transfection with Foxi1 vector or negative control in MKN45/SGC-7901 cells. (**h**) The expression of Wnt3a was determined by qRT-PCR after transfection with Foxi1 vector or negative control in gastric cancer cells. (**i**) MTT assay was performed to test the impact of gastric cancer cells treated with Foxi1 expression vector. (**j**) Colony formation was applied to measure the effect of gastric cancer cells treated with Foxi1 expression vector. (**k**) Apoptosis was conducted to investigate the impact of gastric cancer cells after transfection of Foxi1 expression vector. (**l**) Cell cycle distribution was analyzed by flow cytometry to determine the effect of gastric cancer cells treated with Foxi1 expression vector. (**m**) The expression of pro-caspase 3, active caspase 3, cleaved PARP, BCL-2, CDK6, and CCND1 was detected by western blot (*P<0.05, **P<0.01)

Public databases, including the GEO database, MERAV (http://merav.wi.mit.edu/), and cBioPortal (http://www.cbioportal.org/index.do), showed that Foxi1 is significantly decreased in GC tissues compared to non-tumor adjacent tissues (Supplementary Figure S1). In our present study, the Foxi1 mRNA level was markedly decreased in GC tissues and cell lines (Figures 6a and b). The UCSC genome browser tool, VISTA, and the JASPAR database were applied, and we found a putative Foxi1-binding site located 0.2 kb upstream of the *FOCAD* gene (Figure 6c). When the Foxi1-binding site reporter constructs (including Foxi1-binding site-WT and Foxi1-binding site-MUT) and Foxi1 expression vectors were co-transfected into HEK293 cells, the Foxi1-binding site-WT reporter had higher luciferase activity compared to the mutant reporter (Figure 6d). Consistent with these data, the ChIP experiment indicated the Foxi1 protein binds to the putative binding site upstream of miR-491 (Figures 6e and f). The increased expression level of Foxi1 in MKN45/SGC-7901 cells transfected with the Foxi1 overexpression vector was verified (Supplementary Figure S3D). Accordingly, overexpression of Foxi1 in GC cells led to increased miR-491-5p expression and decreased Wnt3a expression, suggesting there is an axis among Foxi1/miR-491-5p/Wnt3a signaling (Figures 6g and h). In addition, Foxi1 inhibited cell proliferation, and induced apoptosis and cell cycle arrest in GC cells (Figures 6i–k). Furthermore, overexpression of Foxi1 decreased the expression levels of pro-caspase 3, BCL-2, CDK6, and CCND1, but increased the expression level of active caspase 3 and cleaved PARP (Figure 6m), suggesting that Foxi1 contributes to the proliferation inhibition, apoptosis promotion, and cell cycle arrest by modulating miR-491-5p transcription in GC cells.

### Foxi1 mediates the miR-491-5p suppression of GC progression by targeting the Wnt3a/*β*-catenin signaling pathway

**Figure 7:**
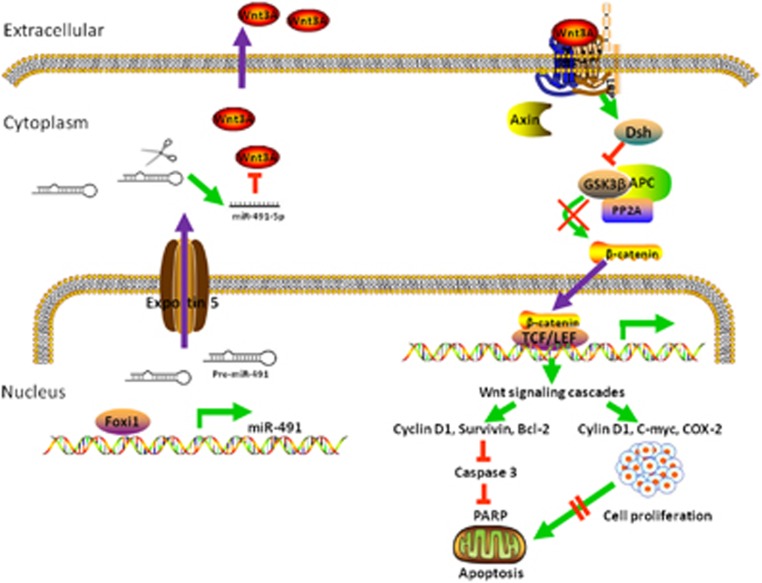
Proposed model for miR-491-5p, mediated by Foxi1, functions as a tumor suppressor by targeting Wnt3a/*β*-catenin signaling in the development of gastric cancer. Foxi1 binds to miR-491 promoter and activates the miR-491-5p expression. miR-491-5p mediates the proliferation inhibition and apoptosis promotion by targeting Wnt3a thereby affects WNT/*β*-catenin signaling and related downstream molecules in gastric cancer cells

Foxi1 binds to the miR-491 promoter sequence and induces the expression of miR-491-5p. Activated miR-491-5p suppresses GC cell progression, and induces cell apoptosis and cell cycle arrest by targeting the Wnt3a/*β*-catenin signaling pathway (Figure 7).

## Discussion

The carcinogenesis of GC is a multifactorial process including genetic and epigenetic events. Emerging evidence indicates that miRNAs are dysregulated in various cancers, and function as tumor suppressors or oncogenes. Numerous studies have characterized the miRNA signatures of GC, but the exact role of miRNA dysregulation in the pathogenesis of GC remains elusive. miR-491-5p was selected as our research subject.

The miR-491-5p gene locates within the fourth intron of *FOCAD* gene. Loss of FOCAD plays a potential tumor suppressor role in breast cancer, colorectal cancer, and glioblastoma.^30, 31, 32^ Recently, miR-491-5p has been reported to be involved in several cancer types.^10, 11, 12, 13^ Until now, no reports have investigated the expression pattern, function, and underlying mechanisms of miR-491-5p in GC. In the current study, miR-491-5p was markedly downregulated in GC tissues. Moreover, our results suggested that miR-491-5p may play an important role in inhibiting GC growth, inhibiting proliferation, disrupting the cell cycle, and inducing apoptosis *in vitro* and *in vivo*. In addition, miR-491-5p altered the expression of the apoptosis/cell cycle-related proteins including caspase 3, PARP, BCL-2, CDK2, and CCNA2. Our data provide a more comprehensive understanding of the tumor suppressor role of miR-491-5p during GC progression.

miRNAs cause mRNA degradation or inhibition by interacting with the 3′-UTR of target gene mRNA. Using bioinformatic analyses and a dual-luciferase reporter assay, we demonstrated that miR-491-5p directly targets Wnt3a by binding its 3′-UTR and inhibiting translation. In addition, our results showed that upregulation of miR-491-5p inhibited the expression of Wnt3a in MKN45/SGC-7901 cancer cells. Wnt3a exerts its function by activating the canonical Wnt signaling pathway followed by transcriptional activation of target genes, such as cyclin D1, C-myc, BCL-2, and survivin. Wnt3a is overexpressed in several cancer types;^18, 19, 20^ a previous study has shown that Wnt3a is highly expressed in human scirrhous gastric carcinoma 44As3 cells with highly metastatic derivatives.^24^ However, to the best of our knowledge, the function of Wnt3a and its clinicopathological significance in human GC has not yet been characterized. Consistent with previous data, our results showed that Wnt3a is upregulated in cancer tissues and downregulated in normal tissues.^24^ To further clarify the tumor suppressor role of miR-491-5p by targeting Wnt3a, siRNA was used to knock down the expression of Wnt3a. Our data showed that Wnt3a silencing inhibited cell proliferation and induced cell apoptosis, which is similar to the effect of miR-491-5p overexpression in GC cells *in vitro*. Moreover, overexpression of Wnt3a substantially reversed the tumor-suppressive effects of miR-491-5p on cell proliferation and apoptosis in a rescue experiment. While, the colony-forming ability and cell cycle distribution were slightly altered by siWnt3a. Accordingly, the expression levels of related proteins, including *β*-catenin, pro-caspase 3, active caspase 3, cleaved PARP, BCL-2, and CCND1, were also altered by siWnt3a. In addition, overexpression of miR-491-5p by lentiviral infection also inhibited tumor growth in nude mice. The expression levels of Wnt3a were downregulated in tumor nodules injected by miR-491 lentivirus-infected cells compared to negative control lentivirus-infected cells, which was similar to the results in GC cell lines. Such studies have also been performed for lung cancer.^6^ Together, these findings suggest that miR-491-5p mechanistically acts as a tumor suppressor via regulation of the Wnt3a/*β*-catenin signaling pathway.

In the current study, bioinformatic analyses suggested that Foxi1 might target the upstream region of miR-491. Furthermore, the GEO database, MERAV, cBioPortal database, and our present study show that the expression of Foxi1 is markedly decreased in GC tissues compared to their normal counterparts. These findings suggest that Foxi1 might be involved in the regulation of miR-491-5p in GC development. In our present study, function analyses showed that Foxi1 inhibited GC cell growth and proliferation. In addition, Foxi1 led to cell cycle arrest and induced cell apoptosis. Moreover, related proteins, including pro-caspase 3, active caspase 3, cleaved PARP, BCL-2, CCND1, and CDK6, were also changed by Foxi1 expression. On the basis of ChIP and luciferase assays, our results confirmed that Foxi1 directly binds to the miR-491 promoter. To the best of our knowledge, this is the first report demonstrating that Foxi1 may function as a tumor suppressor in GC development. Foxi1 belongs to the forkhead-box (FOX) family of genes, which have been implicated in carcinogenesis through gene amplification, chromosomal translocation, retroviral integration, and transcriptional regulation.^33^ The *FOXA1* gene is amplified and overexpressed in lung and esophageal cancer, and it is upregulated in pancreatic cancer and basal cell carcinoma. The *FOXOl* gene is fused to the *PAX3* or *PAX7* gene in rhabdomyosarcoma.^33^ Our data provide new evidence for the carcinogenic mechanism of the FOX family.

There are several new points in the present study. First, our study is the first to report that overexpression of Wnt3a is associated with perineural invasion in GC patients. The underlying mechanism may be due to the activation of Wnt/*β*-catenin signaling downstream of target MMPs.^34^ Previously, it has been suggested that perineural invasion is a poor prognostic marker of GC.^35, 36^ A larger population of samples should be used to confirm if Wnt3a can serve as a predictive marker of GC. Second, this is the first study to report the tumor suppressor role of Foxi1 by transcriptional regulation of miR-491-5p in the progression of GC. Further studies are required to elucidate the role of Foxi1 in the carcinogenic mechanism by gene amplification, chromosomal translocation, or retroviral integration similar to other FOX members.

In conclusion, our data reported the following new findings: (1) miR-491-5p is frequently downregulated in GC tissues and cell lines; (2) miR-491-5p functions as a tumor suppressor in GC cells; (3) Wnt3a is a downstream target gene of miR-491-5p; (4) miR-491-5p inhibits cell proliferation and promotes apoptosis in GC cells by regulating Wnt3a/*β*-catenin signaling; (5) Foxi1 is significantly decreased in GC tissues and acts as a tumor suppressor; (6) Foxi1 binds to the miR-491 promoter and activates its expression; and (7) there is a regulation axis among Foxi1/miR-491-5p/Wnt3a/*β*-catenin signaling during the development of GC. Our study underscores the important role of miR-491-5p in GC progression, and we expect that our findings for the Foxi1/miR-491-5p/Wnt3a/*β*-catenin signaling axis will provide useful information for the development of more effective and promising therapies against GC.

## Materials and methods

### GC tissues and cancer-derived cell lines

In total, 44 gastric tissues were tested in this study, encompassing 22 tumor samples from GC patients and 22 matched adjacent non-malignant tissue samples from the same patients. All the tissues were surgically obtained from the First Affiliated Hospital of Xi'an Jiaotong University. No radiotherapy or chemotherapy was conducted before surgery, and the patients with prior cancer history were excluded from this study. All tissue specimens were snap-frozen in −80 °C after resection until needed. In addition, to investigate the expression and localization of Wnt3a, one tissue microarray including 92 patients with matched non-cancerous tissue was performed. All cases of GC were evaluated histologically by two senior pathologists independently according to Lauren's and the World Health Organization's classifications (IARC Press, Lyon, 2000) and staged using the TNM staging of the International Union Against Cancer (UICC, 2002). This study was approved by the Ethics Committee of Xi'an Jiaotong University, and informed consent was obtained from all individuals. Human GC cell lines, including BGC823 (poorly differentiated), SGC-7901 (poorly differentiated and metastatic), MKN45 (poorly differentiated), and AGS, in addition to the GES-1 non-malignant gastric epithelium cell line were maintained in the Key Laboratory of Environment and Genes Related to Diseases at Xi'an Jiaotong University. All the cells were cultured in RPMI-1640 medium (Thermo Scientific Hyclone, Waltham, MA, USA) supplemented with 10% fetal bovine serum at 37 °C  in a humidified cell incubator with an atmosphere of 5% CO_2_ and were subcultured during their logarithmic phase.

### RNA extraction and quantitative reverse transcription PCR

Total RNA was isolated from the tissues and cells using TRIzol reagent (Invitrogen, Carlsbad, CA, USA) according to manufacturer's instructions. RNA was quantified using a NanoDrop ND-1000 spectrophotometer (NanoDrop Technologies, Wilmington, DE, USA). Total RNA (500 ng) and PrimeScript RT reagent (Takara, Kusatsu, Japan) were used to generate cDNA, which was subjected to qRT-PCR using SYBR Green Master Mix (Takara) performed on a FTC-3000TM System (Funglyn Biotech Inc., Toronto, Ontario, Canada). *β*-actin and small RNA RNU6B (U6) were used as endogenous controls to normalize mRNA and miRNA, respectively. The 2^−ΔΔCT^ method was used to quantify the relative levels of mRNA and miR-491-5p. Each sample was performed in triplicate. All primers used in the present study are shown in Supplementary Table S2.

### Expression vector construction

The miR-491 (pre-miR-491) expression vector and related control vector were constructed by synthetic oligonucleotides and cloned into the pcDNA6.2-GW/EmGFP vector (Invitrogen) between the EcoRI and HindIII sites as previously described.^37, 38^ Using bioinformatic analyses, we identified a fragment of Wnt3a as a miR-491-5p target. The 3′-UTR of the human Wnt3a mRNA was constructed by synthetic oligonucleotides and cloned into the pmir GLODual-Luciferase miRNA Target Expression Vector (Promega, Madison, WI, USA) between the SacI and XhoI sites as previously described.^37, 38^ A miR-491-5p inhibitor, an inhibitor negative control, and siRNAs targeting Wnt3a were chemically synthesized (GenePharma, Shanghai, China). Wnt3a was cloned into the GV230 vector (Genechem, Shanghai, China) between the XhoI and KpnI sites. Foxi1 was cloned into PEGFP-C1 between the HindIII and BamHI sites (GENEWIZ, Suzhou, China). A 127 bp sequence containing the wild-type or mutant Foxi1-binding site was cloned into the pGL3 promoter vector between the KpnI and XhoI sites (GENEWIZ). All the sequence information is given in Supplementary Table S2.

### Oligonucleotide and plasmid transfection

JetSI-ENDO transfection reagents (Polyplus-transfection, Strasbourg, France) were used for cell transfection according to manufacturer's instructions. The transfection efficiency was verified by quantitative real-time PCR. The controls were used under the same experimental conditions as the miRNA mimics, thus allowing a transfection efficiency assessment during each assay.

### Cell proliferation assay

MKN45 and SGC-7901 cells (4000 cells per well) were seeded into 96-well plates with 100 *μ*l of RPMI-1640 and incubated for 24, 48, and 72 h. Before the indicated time, 20 *μ*l of MTT reagent (Sigma, St. Louis, MO, USA) was added to each well, and the cells were incubated for additional 4 h. The culture medium was then removed and 100 *μ*l of DMSO (Sigma) was added. Cell viability was assessed using the 3-(4, 5-dimethyl-2-thiazolyl)-2,5-diphenyl-2H-tetrazolium bromide (MTT) assay FLUO star OPTIMA (BMG LABTECH, Offenburg, Germany) by measuring the absorbance at 490 nm. The absorbance is correlated with the number of viable cells. Each experiment contained three replicates, and the mean values and S.D. were calculated.

### Colony formation assay

For the colony formation assay, MKN45 and SGC-7901 (2000 cells per well) were seeded into six-well plates 24 h after transfection. After 10 days of incubation, the plates were gently washed with phosphate-buffered saline (PBS), and the colonies were stained with 0.1% crystal violet for 30 min after fixation with methanol for 10 min. Excess dye was rinsed off by PBS. Images of the colonies were acquired, and the colonies were counted using computer software (Quantity One, Bio-Rad, Hercules, CA, USA).

### Cell apoptosis assay

In brief, MKN45 and SGC-7901 cells were seeded into 12-well plates at a density of 1 × 10^6^ cells per well in triplicate, and 48 h after transfection, cells were collected and stained using the Annexin V-FITC/PI Apoptosis Detection Kit (KeyGEN BioTECH, Nanjing, China) according to manufacturer's instructions. Early and late cell apoptosis events were examined via flow cytometry ((FACSort), Becton Dickinson, San Jose, CA, USA), and apoptotic cells were described as a percentage by ModFit software (Verity Software House, Augusta, ME, USA).

### Cell cycle assay

In short, MKN45 and SGC-7901 cells were seeded into 12-well plates with a density of 1 × 10^6^ cells per well, and 48 h after transfection, cells were collected by trypsinization, washed with PBS and fixed in ice-cold 70% ethanol alcohol at 4 °C overnight. The cells were then washed with PBS again and incubated in 0.1 mg/ml RNase A and 0.05 mg/ml propidium iodide for 30 min at 4 °C. Cell distributions in G0–G1, S, and G2–M phases were assayed by flow cytometry (FACSort; Becton). Independent experiments were performed in triplicate.

### Orthotropic tumor model in nude mice

Tumorigenicity assays in animals were conducted according to the guidelines of the Animal Care and Use Committee of Xi'an Jiaotong University. Four-week-old male nude mice (BALB/c-nude) were used to examine tumorigenicity. In brief, SGC-7901 cells were transfected with LV-GFP-miR-491 or LV-GFP-miR-negative control lentiviral vectors (Genechem), and 1 × 10^6^ miR-491 and miR-negative control cells were separately injected into each mouse armpit, thus generating tumor comparisons within each mouse. Tumor size was measured every 3 days. Eight weeks after injection, mice were killed and tumor nodules were weighed. The tumor volume (*V*) was calculated as follows: *V*=(*a* × *b*^2^)/2; where *a* and *b* are the long and short axes of the tumor nodule, respectively. For end point experiments, the animals were anesthetized by isoflurane as described previously, and the tumors in living mice were measured by observing GFP expression using a photobiology system (Xenogen IVIS Spectrum imaging system (Xenogen, Alameda, CA, USA)).

### Western blot analysis

Briefly, 48 h after transfection, protein was extracted using radioimmunoprecipitation assay cell lysis buffer (Wolsen, Xi'an, China), and protein concentration was determined by a BCA protein assay kit (Thermo Scientific Pierce, Beijing, China). Equal amounts of protein lysates were separated by 10% SDS polyacrylamide gels and electrophoretically transferred to a methanol-activated polyvinylidene difluoride membrane (Millipore, Billerica, MA, USA). After blocking in 5% non-fat dry milk in Tris-buffered saline (pH 7.4) containing 0.1% Tween (TBST) for 1 h, the membranes were incubated with the following primary antibodies overnight at 4 °C: Wnt3a (Bioss, Beijing, China, diluted 1:200), *β*-catenin (Cell Signaling Technology, diluted 1:1000), Caspase 3 (Cell Signaling Technology, Danvers, MA, USA; diluted 1:1000), active caspase 3 (ProteinTech Group, Wuhan, China, diluted 1:1000), cleaved PARP (Abcam, Cambridge, MA, USA; diluted 1:1000), BCL-2 (ProteinTech Group, diluted 1:1000), CDK2 (Cell Signaling Technology, diluted 1:1000), CCNA2 (Cell Signaling Technology, diluted 1:1000), CCND1 (ProteinTech Group, diluted 1:1000), CDK6 (Cell Signaling Technology, diluted 1:1000), Foxi1 (Abcam, diluted 1:1000), and *β*-actin (Santa Cruz Biotechnology, Santa Cruz, CA, USA; diluted 1:2000). After washing three times with TBST, the membrane was incubated with goat anti-rabbit or goat anti-mouse horse-radish peroxidase (HRP)-conjugated secondary antibodies (1:1000, Santa Cruz Biotechnology) for 1 h at room temperature. The expression levels of the proteins were normalized to *β*-actin levels in each sample. Immunoreactive bands were visualized using Western Lightning Chemiluminescence Reagent Plus (PerkinElmer, Waltham, MA, USA) in accordance with manufacturer's instructions. The blots were scanned, and the band density was then quantified by Quantity One imaging software (Bio-Rad, Hercules, CA, USA).

### Immunohistochemistry

IHC was performed as described previously.^39^ Tissue microarray sections were deparaffinized with xylene and hydrated using an alcohol gradient. Endogenous peroxidase-blocking and antigen retrieval were performed sequentially. The sections were then incubated with polyclonal rabbit anti-Wnt3a (Bioss, diluted 1:100) followed by incubation with secondary antibody conjugated with HRP. Detection was conducted by 3,3′-diaminobenzidine and hematoxylin. The degree of immunostaining was evaluated by two independent pathologists who were blind to the clinical data of the patients. We used a scoring standard for Wnt3a protein expression, and both the percentage of positive cells and intensity were considered as previously described.

### Dual-luciferase assay

HEK293 cells were seeded into 96-well plates (10 000 cells per well), and 100 ng of pmirGLO-Wnt3a-3′-UTR vectors, including wild-type or mutated miR-491-5p-binding sites, was co-transfected with 100 ng of pre-miR-491 expression vector into HEK293 cell lines, and pmirGLO vector was used as the control. In a second experiment, the Foxi1 expression vector (or empty vector) was co-transfected with wild-type or mutated Foxi1-binding site reporter constructs, and a blank pGL3 luciferase vector was used as a positive control. Twenty-four hours after transfection, firefly and Renilla luciferase activity was examined using the Dual-Luciferase Assay System (Promega). The relative expression of firefly luciferase activity was normalized to Renilla luciferase activity. Each assay was performed three times.

### TCF/LEF reporter assay

The pGL4.49 (luc2P/TCF–LEF/Hygro; 0.1 *μ*g per well) vector containing eight copies of a TCF–LEF response element that drives transcription of the luciferase reporter gene *luc2P* (*Photinus pyralis*) was co-transfected with either miR-control, miR-491, and miR-491+Wnt3a vectors into HEK293 cells in a 96-well plate. Forty-eight hours post transfection, firefly luciferase activity was examined using the ONE-Glo Luciferase Assay System (Promega). Luminescence was detected in a 1420-Multilabel counter. For each assay, five independent replicates were performed. Each assay was performed three times.

### Chromatin immunoprecipitation assay

The binding of Foxi1 to the miR-491 promoter was evaluated using chromatin immunoprecipitation (ChIP) analysis. Briefly, protein/DNA complexes obtained from SGC-7901 cells were cross-linked using 1% formaldehyde (Bio-Rad, Hercules, CA, USA) for 15 min at room temperature, and the reactions were quenched by adding glycine (0.125 M) for 30 min. The cells were rinsed two times with 5 ml of PBS. Cells were collected, and the nuclei were resuspended in Mg-NI, Mg-NIXP40, Ca-NI (0.5 M EGTA addition), and lysis buffer (with protease inhibitors). The samples were then sonicated, and the chromatin was sheared into an average length of ~100–500 bp. The insoluble material was removed by centrifugation at 14 000 × *g*. Of the DNA/protein complexes, 100 *μ*l was used as input for ChIP. Foxi1 was immunoprecipitated from the supernatant using an anti-Foxi1 antibody (normal rabbit IgG antibodies were used as negative controls) at 4 °C for 4 h and bound to protein G sepharose (Invitrogen) during a 2 h incubation at 4 °C. The immunoprecipitates were then washed two times successively with the following solutions: ChIP lysis buffer, ChIP lysis buffer containing 500 mM NaCl, LiCl/detergent solution (10 mM Tris–HCl, pH 8.0; 250 mM LiCl, 0.5% NP-40, 0.5% sodium deoxycholate, and 1 mM EDTA), and finally TE buffer (10 mM Tris and 1 mM EDTA, pH 8.0). A solution containing 1% SDS and 0.1 M sodium bicarbonate was used to elute the bound proteins from beads. The input and the eluent samples were reverse cross-linked with proteinase K by incubating at 65 °C for 8 h. Phenol/chloroform (Invitrogen) was used to isolate the DNA from the samples followed by ethanol precipitation. Promoter binding was determined by PCR with primers spanning the miR-49 upstream regions and SYBR Green Master Mix (Takara) using a LightCycler 480 (Roche Diagnostics, Tokyo, Japan). Enrichments were calculated as percentage of the input. The primer sequence oligonucleotides used for PCR amplification are shown in Supplementary Table S2.

### Statistical analysis

All data are presented as mean±S.D. of at least three independent experiments. Differences between two groups or among three groups were analyzed by Student's *t*-test or one-way ANOVA. The linear correlation coefficient (Pearson *r*) was calculated to estimate the correlation between miR-491-5p values and Wnt3a levels in the matched GC tissues. The relationship between Wnt3a expression level and clinical parameters was calculated by the *χ*^2^-test. Data were considered to be statistically significant when *P*-values were <0.05. All of the data were analyzed using SPSS13.0 software (SPSS Inc., Chicago, IL, USA). All tests were two-sided, and differences were considered statistically significant at *P*<0.05.

## Figures and Tables

**Table 1:** Clinicopathologic characteristics of patients according to Wnt3a expression

**Variables**	**Wnt3a negative (N, %)**	**Wnt3a positive (N, %)**	**χ*^2^***	***P*-value**
*Gender*				
Male	21 (84.0)	24 (52.2)	7.069	0.010*
Female	4 (16.0)	22 (47.8)		
				
*Age (years)*				
Median, range (64, 24–84)				
<60	12 (48.0)	21 (47.7)	1.119	0.681
⩾60	13 (52.0)	23 (52.2)		
				
*Location*				
Upper	9 (36.0)	11 (25.0)	2.834	0.481
Middle	1 (4.0)	5 (11.4)		
Down	15 (60.0)	28 (63.6)		
				
*Tumor size (mm)*				
<5 cm	9 (36.0)	14 (30.4)	0.229	0.791
⩾5 cm	16 (64.0)	32 (69.6)		
				
*Histologic type*				
Tubular adenocarcinoma	11 (44.0)	15 (32.6)	1.017	0.577
Mucinous adenocarcinoma	8 (32.0)	15 (32.6)		
Signet-ring cell carcinoma	6 (24.0)	16 (34.8)		
				
*T stage*				
T1	0 (0)	2 (6.9)	6.714	0.200
T2	1 (5.3)	2 (6.9)		
T3	14 (73.6)	23 (79.3)		
T4A	3 (15.8)	0 (0)		
T4B	1 (5.3)	2 (6.9)		
				
*N stage*				
N0	6 (26.1)	9 (22.0)	1.948	0.885
N1	3 (13.0)	9 (22.0)		
N2	6 (26.1)	12 (29.3)		
N3A	7 (30.4)	8 (19.5)		
N3B	1 (4.3)	3 (7.3)		
				
*Metastasis*				
Negative	25 (100.0)	45 (97.8)	0.551	1.000
Positive	0 (0)	1 (2.2)		
				
*pTNM stage*				
IA	0 (0)	1 (4.2)	7.337	0.496
IB	1 (5.9)	0 (0)		
IIA	4 (23.5)	5 (20.8)		
IIB	3 (17.6)	4 (16.7)		
IIIA	3 (17.6)	9 (37.5)		
IIIB	5 (29.4)	3 (12.5)		
IIIC	1 (5.9)	2 (83.3)		
IV	0 (0)	0 (0)		
				
*Regional lymphatic invasion*				
Negative	6 (25.0)	13 (28.3)	0.085	0.788
Positive	18 (75.0)	33 (71.7)		
				
*Perineural invasion*				
Negative	25 (100.0)	37 (80.4)	5.601	0.022*
Positive	0 (0)	9 (19.6)		
