# Supplementary material for: miR-491-5p, mediated by Foxi1, functions as a tumor suppressor by targeting Wnt3a/β-catenin signaling in the development of gastric cancer
Source: Cell Death Dis. 2017 Mar 30;8(3):e2714–. doi: 10.1038/cddis.2017.134 (PMC5386537; doi:10.1038/cddis.2017.134)
Supplement: Supplementary Information [file cddis2017134x1.docx]

**Supplementary Table S1 Clinicopathologic characteristics of patients with gastric cancer**

| **Variables** | **Patients (n, %)** |
| --- | --- |
| **Gender** |  |
| Male | 56 (60.8) |
| Female | 36 (39.1) |
| **Age (years)** |  |
| median, range (64,24-84) |  |
| ＜60 | 43 (65.2) |
| ≥60 | 23 (34.8) |
| **Location** |  |
| Upper | 22 (24.4) |
| Middle | 8 (8.9) |
| Down | 60 (66.7) |
| **Tumor size (mm)** |  |
| ＜5cm | 29 (32.2) |
| ≥5cm | 61 (67.8) |
| **Histologic type** |  |
| Tubular adenocarcinoma | 30 (32.6) |
| Mucinous adenocarcinoma | 31 (33.7) |
| Signet ring cell carcinoma | 31 (33.7) |
| **T stage** |  |
| T1 | 2 (3.4) |
| T2 | 4 (6.9) |
| T3 | 45 (77.6) |
| T4A | 4 (6.9) |
| T4B | 3 (5.2) |
| **N stage** |  |
| N0 | 19 (22.4) |
| N1 | 19 (22.4) |
| N2 | 22 (25.9) |
| N3A | 20 (23.5) |
| N3B | 5 (5.9) |
| **Metastasis** |  |
| Negative | 89 (96.7) |
| Positive | 3 (3.3) |
| **pTNM stage** |  |
| IA | 1 (1.9) |
| IB | 1 (1.9) |
| IIA | 11 (21.2) |
| IIB | 7 (13.5) |
| IIIA | 15 (28.8) |
| IIIB | 11 (21.1) |
| IIIC | 3 (5.8) |
| IV | 3 (5.8) |
| **Regional lymphatic invasion** |  |
| Negative | 23 (25.0) |
| Positive | 69 (75.0) |
| **Perineural invasion** |  |
| Negative | 79 (85.9) |
| Positive | 13 (14.1) |
| **Wnt3a expression** |  |
| Negative | 25 (35.2) |
| Positive | 46 (64.8) |

**Supplementary Table S2. Primers and oligonucleotides used in this work**

| **Name** | **Sequence 5'-3'** |
| --- | --- |
| Pre-miR-491-S | AATTCTTGACTTAGCTGGGTAGTGGGGAACCCTTCCATGAGGAGTAGAACACTCCTTATGCAAGATTCCCTTCTACCTGGCTGGGTTGGA |
| Pre-miR-491-AS | AGCTTCCAACCCAGCCAGGTAGAAGGGAATCTTGCATAAGGAGTGTTCTACTCCTCATGGAAGGGTTCCCCACTACCCAGCTAAGTCAAG |
| miR-491-5p-inhibitor ctrl | CAGTACTTTTGTGTAGTACAA |
| miR-491-5p-inhibitor | CCTCATGGAAGGGTTCCCCACT |
| Wnt3a 3′UTR-S | CCCCTGCCCTCGGGTCTCCCCACCC |
| Wnt3a 3′UTR-AS | TCGAGGGTGGGGAGACCCGAGGGCAGGGGAGCT |
| Wnt3a 3′UTR-MUT-S | CCCCTGCCCTCGGGTCTCCGGTCCC |
| Wnt3a 3′UTR-MUT-AS | TCGAGGGACCGGAGACCCGAGGGCAGGGGAGCT |
| si-ctrl-S | UUCUUCGAACGUGUCACGUTT |
| si-ctrl-AS | ACGUGACACGUUCGGAGAATT |
| siWnt3a-S | CCCACUCGGAUACUUCUUATT |
| siWnt3a-AS | UAAGAAGUAUCCGAGUGGGTT |
| U6-RT | CGCTTCACGAATTTGCGTGTCAT |
| U6-F | GCTTCGGCAGCACATATACTAAAAT |
| U6-R | CGCTTCACGAATTTGCGTGTCAT |
| miR-491-5p-RT | GTCGTATCCAGTGCGTGTCGTGGAGTCGGCAATTGCACTGGATACGACCCTCATG |
| miR-491-5p-F | ATCCAGTGCGTGTCGTG |
| miR-491-5p-R | TGCTAGTGGGGAACCCTTC |
| β-actin-F | CGGGAAGCTTGTCATCAATGG |
| β-actin-R | GGCAGTGATGGCATGGACTG |
| Wnt3a-F | AACTGCACCACCGTCCAC |
| Wnt3a-R | AAGGCCGACTCCCTGGTA |
| Foxi1-F | TCCCCTTCTACAACAAGAGCA |
| Foxi1-R | AGGGTCCAGTAATTCCCTTTG |
| Foxi1 binding site primer-F | GGACACTTAAGTCTGCAGAGGTTAC |
| Foxi1 binding site primer-R | AGTCTGAGATCAACCTGCAAGGCGG |

**Figure lengeds**

**Supplementary Figure S1 The inhibitory effect of miR-491-5p inhibitor on GES-1 cells**

**A**. MKN45/SGC-7901 cells were transfected with miR-491 vector or control vector, qRT-PCR was performed to examine miR-491-5p expression, U6 was served as a internal control. **B**. MTT assay of GES-1 cells after transfection with ASO-miR-491 or a negative control. **C**. Apoptosis was determined in GES-1 cells transfected with ASO-miR-491 or a negative control. **D**. Cell cycle was determined in GES-1 cells transfected with ASO-miR-491 or a negative control. **E**. The expression of pro-caspase 3, active caspase 3, cleaved-PARP, BCL-2, CDK2, CCNA2, β-catenin was analyzed by western blot (* P < 0.05, ** P < 0.01).

**Supplementary Figure S2 The dual luciferase assay and TCF/LEF reporter assay in MKN45 and SGC-7901 cells**

**A**. The luciferase assay was performed in MKN45 and SGC-7901 cells in which miR-491 was co-transfected with pGLO-Wnt3a wild type or pGLO-Wnt3a mutant vector. **B**. The TCF/LEF reporter luciferase assay was performed into MKN45 and SGC-7901 cells in which pGL4.49 vector containing a TCF-LEF response element was co-transfected with miR-control, miR-491, miR-491+Wnt3a vector (* P < 0.05, ** P < 0.01).

**Supplementary Figure S3 The expression of Foxi1 in gastric cancer tissues and cells**

**A**. The expression of Foxi1 in gastric cancer by using cBioPortal database; **B**. The expression of Foxi1 in gastric cancer by using MERAV database; **C**. The expression of Foxi1 in gastric cancer by using GEO database. **D**. The expression of Foxi1 was analyzed by qRT-PCR and western blot after transfection with Foxi1 vector or empty vector in MKN45/SGC-7901 cells (*: P < 0.05, **: P < 0.01).
