# Supplementary figures and images for: miR-491-5p, mediated by Foxi1, functions as a tumor suppressor by targeting Wnt3a/β-catenin signaling in the development of gastric cancer
Source: Cell Death Dis. 2017 Mar 30;8(3):e2714–. doi: 10.1038/cddis.2017.134 (PMC5386537; doi:10.1038/cddis.2017.134)

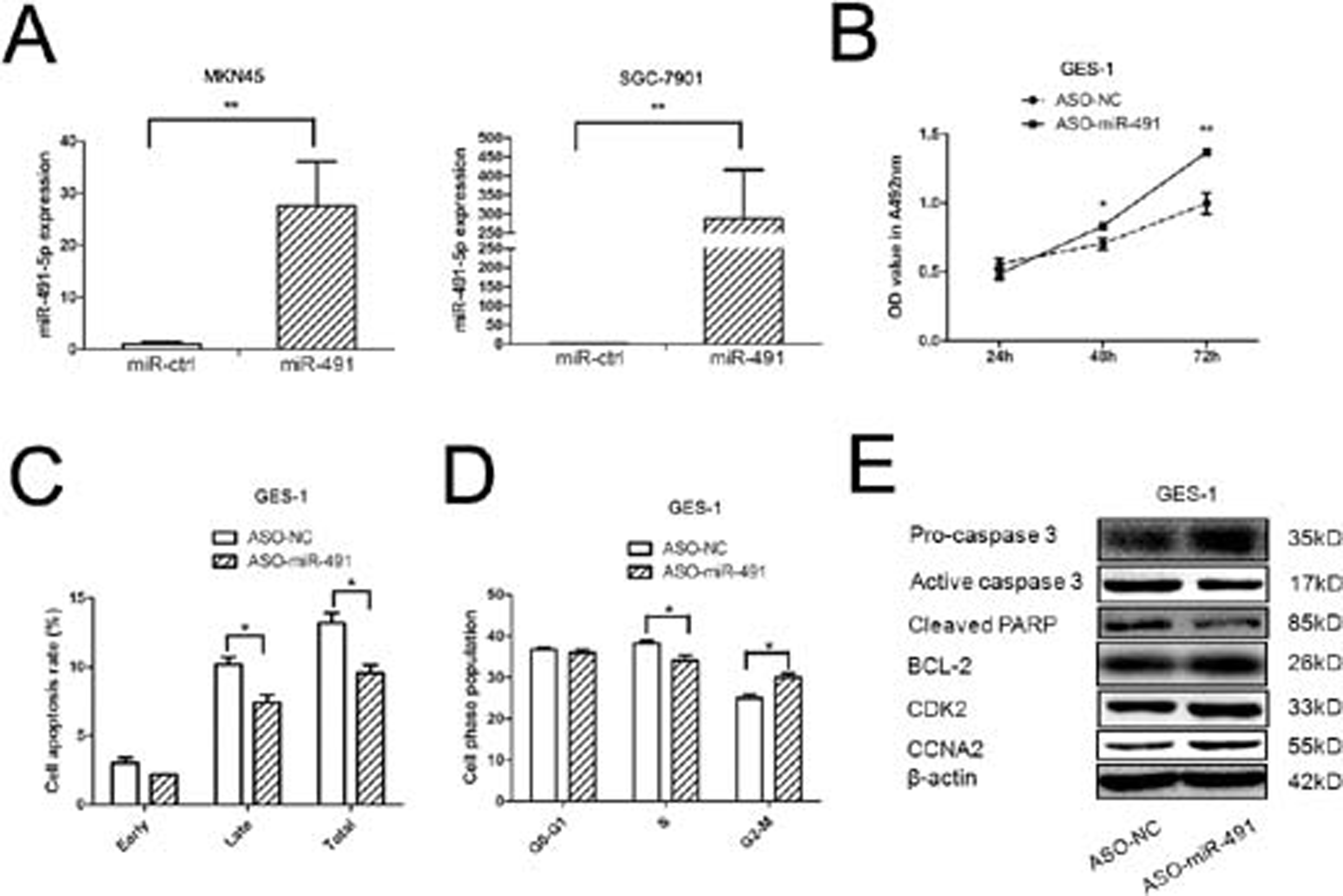

Supplement: Supplementary Figure 1 [file cddis2017134x2.tif]

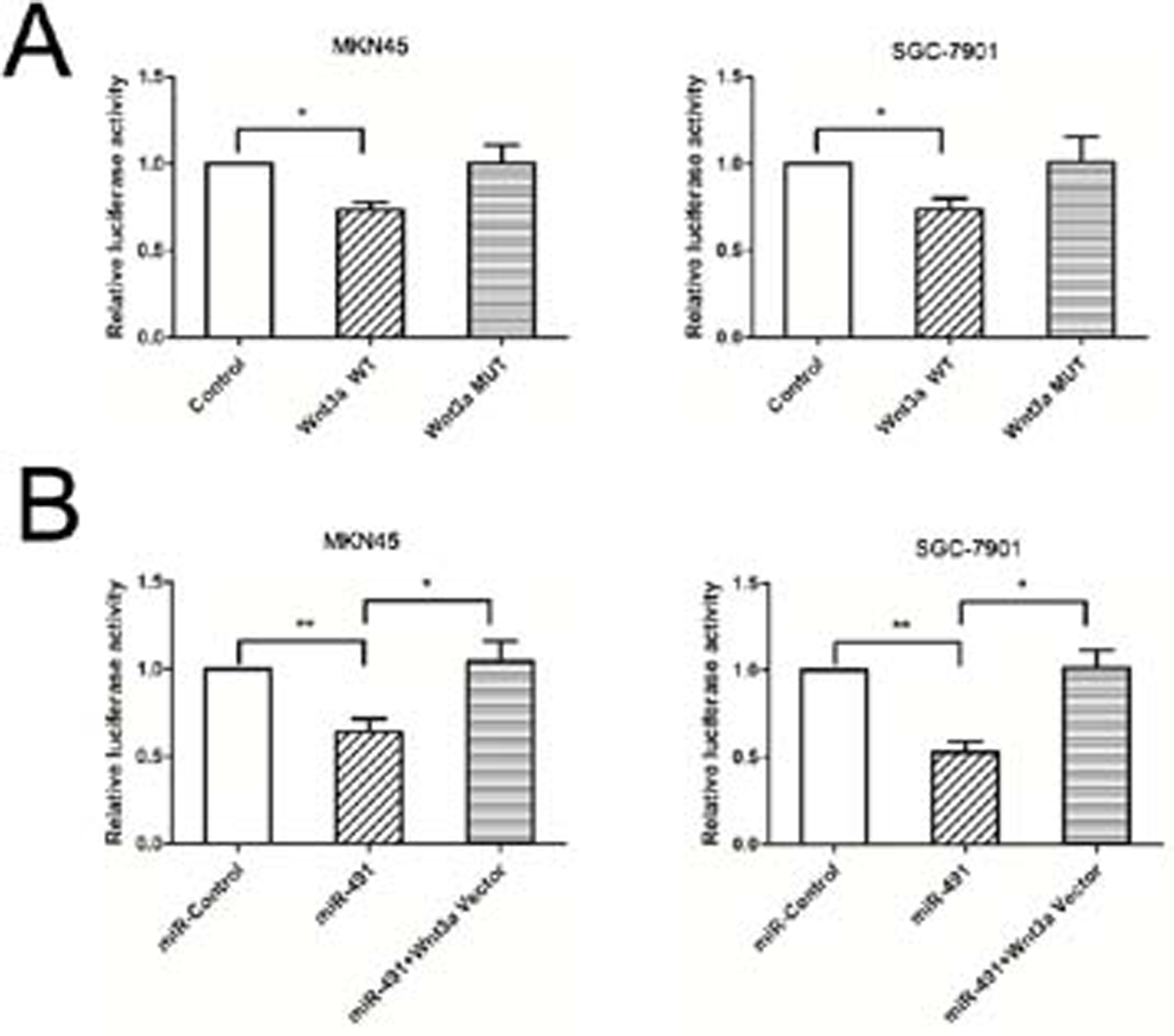

Supplement: Supplementary Figure 2 [file cddis2017134x3.tif]

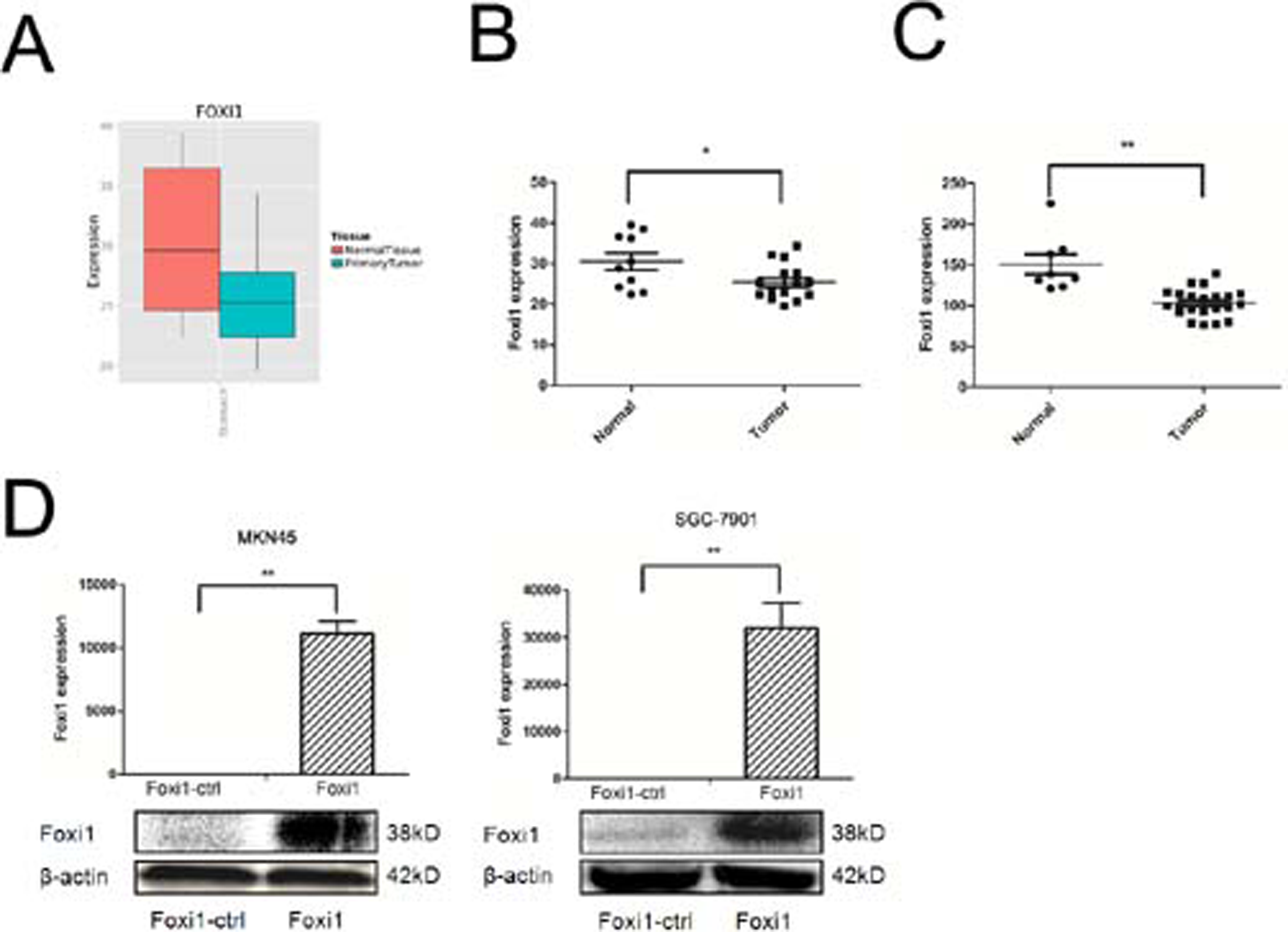

Supplement: Supplementary Figure 3 [file cddis2017134x4.tif]
